# Severe Aortic Stenosis and Regurgitation in a Young Athletic Doctor With Bicuspid Aortic Valve Managed With On-X® Mechanical Valve Replacement

**DOI:** 10.7759/cureus.95274

**Published:** 2025-10-23

**Authors:** Inderjeet Singh, Akash Xavier, Jayant Bajaj

**Affiliations:** 1 Internal Medicine, Southern Health and Social Care Trust, Craigavon, GBR; 2 Emergency Medicine, Southern Health and Social Care Trust, Craigavon, GBR; 3 General Surgery, Dr. D. Y. Patil Medical College, Hospital & Research Centre, Pune, IND

**Keywords:** aortic regurgitation, mechanical aortic valve replacement, on-x aortic valve, ross procedure, severe asymptomatic aortic stenosis, warfarin, young patients

## Abstract

We present the case of a 28-year-old athletic male physician with asymptomatic bicuspid aortic valve (BAV), diagnosed with severe aortic stenosis (AS) and moderate aortic regurgitation (AR) following self-auscultation prompted by nocturnal palpitations and chest vibrations. Echocardiography and computed tomography (CT) confirmed severe AS (peak gradient 79 mmHg, aortic valve area 0.6 cm²), moderate AR, and left ventricular hypertrophy. Due to an aortic annulus size mismatch, the Ross procedure was deemed unsuitable, and an On-X® (Artivion, Inc., Kennesaw, Georgia, United States) mechanical aortic valve was implanted. Postoperative echocardiography demonstrated a peak gradient of 11 mmHg and an ejection fraction of 55%. The patient was discharged on warfarin, aspirin, statins, beta-blockers, and calcium channel blockers with counseling on anticoagulation management. This case highlights the challenges of managing BAV in young, active patients and the role of mechanical valve replacement when anatomical constraints preclude other options.

## Introduction

Bicuspid aortic valve (BAV) represents the most prevalent congenital heart defect, affecting approximately 1-2% of the general population and demonstrating a strong male predominance with a 2:1 ratio [1,2]. This congenital anomaly is characterized by the presence of only two functional cusps instead of the normal three, resulting from incomplete separation of two cusps during embryogenesis [3]. BAV is frequently associated with various complications throughout a patient's lifetime, including progressive aortic stenosis (AS), aortic regurgitation (AR), infective endocarditis, aortic root dilatation, and ascending aortic aneurysm formation [1,4].

The natural history of BAV reveals that approximately 50-60% of patients will require surgical intervention by the sixth decade of life, with AS being the most common indication for surgery in younger patients [5]. Progressive valve dysfunction in BAV typically manifests earlier than in tricuspid aortic valves, with severe AS developing at a median age of 40-50 years, compared to 65-70 years in patients with tricuspid aortic valves [6]. The accelerated degeneration is attributed to abnormal flow patterns and increased mechanical stress on the bicuspid valve structure [7].

Surgical management of severe AS and AR in young adults presents unique challenges and requires careful consideration of multiple factors, including valve durability, anticoagulation requirements, lifestyle considerations, and potential for future interventions [8]. Available surgical options include mechanical valve replacement, bioprosthetic valve replacement, valve repair techniques, and the Ross procedure (pulmonary autograft replacement) [2,9]. The choice of intervention becomes particularly complex in young, physically active patients, where long-term durability and maintenance of an active lifestyle are paramount considerations.

Recent advances in mechanical valve technology, particularly the development of the On-X® mechanical valve (Artivion, Inc., Kennesaw, Georgia, United States), have provided new options for young patients with BAV [10]. The On-X valve offers improved hemodynamic performance, reduced thrombogenicity, and potentially lower anticoagulation requirements compared to traditional mechanical valves [10]. This case describes the comprehensive diagnosis and management of severe AS and moderate AR in a 28-year-old athletic physician, emphasizing the decision-making process for valve replacement and highlighting the role of the On-X mechanical valve in young patients with BAV.

## Case presentation

A 28-year-old male physician with an active athletic lifestyle and no significant prior medical history presented with a several-week history of nocturnal palpitations and an unusual sensation of chest vibrations, particularly noticeable when lying in the prone position. Given his medical background, he performed self-auscultation in January 2025 and identified a moderate-intensity ejection systolic murmur in the aortic area with radiation to the carotid arteries. Recognizing the potential clinical significance of these findings, he promptly sought consultation with a senior cardiologist.

Initial clinical examination confirmed the presence of a grade 3/6 ejection systolic murmur best heard at the right upper sternal border with radiation to the neck. There were no signs of heart failure, and the patient remained completely asymptomatic for exertional dyspnea, chest pain, or syncope despite the concerning cardiac findings. The cardiologist recommended comprehensive echocardiographic evaluation to characterize the underlying pathology.

**Figure 1:**
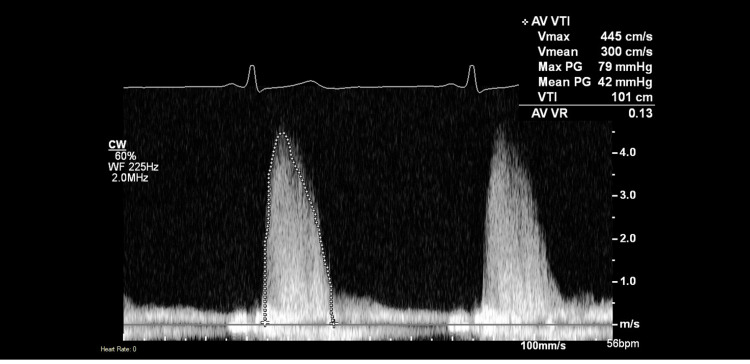
Preoperative TTE With Continuous Wave Doppler Demonstrating Severe Aortic Stenosis. The spectral Doppler waveform shows a characteristic dense, dagger-shaped envelope with markedly elevated transvalvular velocities. Hemodynamic parameters include peak aortic jet velocity (V_max_) of 445 cm/s, mean velocity (V_mean_) of 300 cm/s, peak pressure gradient of 79 mmHg, mean pressure gradient of 42 mmHg, and velocity time integral (VTI) of 101 cm. Minimal aortic regurgitation is present with a valve regurgitation (VR) ratio of 0.13. These findings meet diagnostic criteria for severe, high-gradient aortic stenosis (mean gradient >40 mmHg, peak velocity >4 m/s) and provide a clear indication for surgical aortic valve replacement in this young, symptomatic patient with bicuspid aortic valve disease. TTE: transthoracic echocardiography

Transthoracic echocardiography (TTE) revealed a bicuspid aortic valve with severe aortic stenosis. Continuous wave Doppler interrogation demonstrated markedly elevated transvalvular velocities with a peak velocity (Vmax) of 445 cm/s, mean velocity (Vmean) of 300 cm/second, peak pressure gradient of 79 mmHg, and mean pressure gradient of 42 mmHg (Figure 1). The velocity time integral (VTI) measured 101 cm, and the estimated aortic valve area by the continuity equation was 0.6 cm². Additional findings included aortic regurgitation with a valve regurgitation (VR) ratio of 0.13, left ventricular hypertrophy, and an aortic annulus measurement of 28 mm. These hemodynamic parameters met diagnostic criteria for severe, high-gradient aortic stenosis (mean gradient >40 mmHg, peak velocity >4 m/s), providing a clear indication for surgical intervention.

To further characterize the valve anatomy, transesophageal echocardiography (TEE) was performed, which confirmed an aortic annulus of 30 mm and provided enhanced visualization of the bicuspid valve morphology with evidence of commissural fusion.

Given the importance of accurate anatomical quantification for surgical planning, cardiac CT aortography was subsequently performed. This multimodality imaging approach was essential for comprehensive preoperative assessment, as CT offers superior spatial resolution and direct visualization of valve structure compared to echocardiography, which can be limited by acoustic shadowing and geometric assumptions in valve area calculations. CT imaging demonstrated cusp thickening with dense calcifications, prominent cardiac size secondary to left ventricular hypertrophy, and normal ascending aortic dimensions. CT-derived measurements confirmed the aortic annulus dimensions at 30 mm and, critically, revealed a pulmonary annulus of 24 mm. This significant annulus size mismatch (6 mm discrepancy) between the aortic and pulmonary annuli represented a relative contraindication to the Ross procedure, as it would likely result in suboptimal hemodynamic performance and increased risk of early autograft dysfunction.

Given the patient's medical knowledge and understanding of surgical options, he initially expressed a strong preference for the Ross procedure to avoid the need for lifelong anticoagulation and preserve his active lifestyle without warfarin-related restrictions. However, after detailed discussion of the imaging findings and surgical considerations, it was explained that the substantial annulus mismatch (aortic annulus 30 mm versus pulmonary annulus 24 mm) made this approach technically unsuitable and carried an unacceptably high risk of progressive autograft dilatation and valvular insufficiency.

Following multidisciplinary team discussion involving cardiothoracic surgeons, cardiologists, and the patient, the decision was made to proceed with mechanical aortic valve replacement. In April 2025, aortic valve replacement with a 25 mm On-X mechanical bileaflet valve was performed via median sternotomy under cardiopulmonary bypass. The severely stenotic and calcified bicuspid aortic valve was excised, and the mechanical prosthesis was implanted in the supra-annular position. The procedure was uncomplicated, with cardiopulmonary bypass time of 78 minutes and aortic cross-clamp time of 52 minutes. The patient had an uneventful postoperative recovery and was discharged on postoperative day eight.

**Figure 2:**
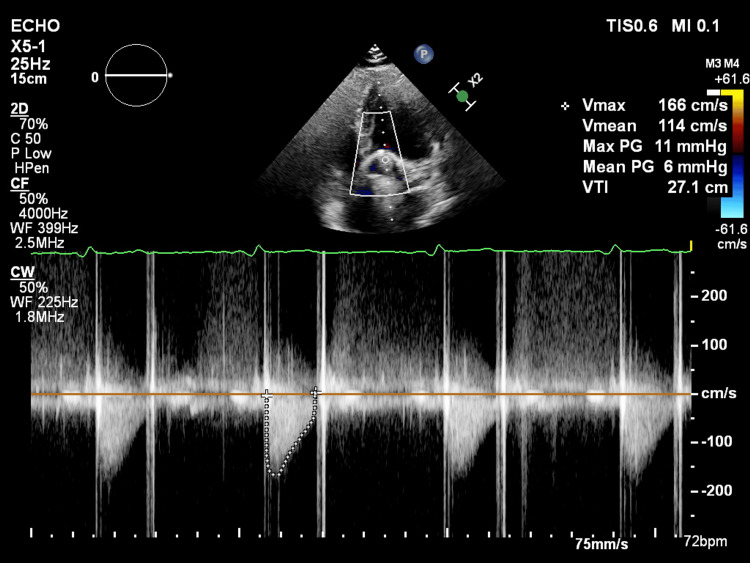
Postoperative TTE With Continuous Wave Doppler Showing Normal Prosthetic Valve Hemodynamics. The upper panel shows two-dimensional imaging demonstrating the prosthetic valve in the aortic position with characteristic acoustic shadowing. The lower panel displays the continuous wave Doppler spectral tracing across the prosthetic valve, revealing excellent hemodynamic performance with a peak velocity (V_max_) of 166 cm/s, mean velocity (V_mean_) of 114 cm/s, peak pressure gradient of 11 mmHg, mean pressure gradient of 6 mmHg, and velocity time integral (VTI) of 27.1 cm. The smooth Doppler envelope without turbulence indicates normal prosthetic valve function with no evidence of stenosis, patient-prosthesis mismatch, or significant regurgitation. These findings represent a dramatic improvement from preoperative values (peak gradient 79 mmHg, mean gradient 42 mmHg) and confirm successful mechanical aortic valve replacement with optimal hemodynamic outcomes. TTE: transthoracic echocardiography

Postoperative transthoracic echocardiography performed five months after surgery demonstrated excellent prosthetic valve function (Figure 2). Continuous wave Doppler assessment revealed normal transprosthetic hemodynamics with a peak velocity of 166 cm/second, mean velocity of 114 cm/second, peak pressure gradient of 11 mmHg, mean pressure gradient of 6 mmHg, and VTI of 27.1 cm. These low gradients confirmed optimal valve function without stenosis or patient-prosthesis mismatch, representing an 86% reduction in mean gradient compared to preoperative values. The smooth Doppler spectral envelope without turbulence indicated unobstructed flow, and no significant paravalvular or transvalvular regurgitation was identified.

The patient was initiated on warfarin therapy with a target international normalized ratio (INR) of 2.0-3.0 for the mechanical aortic valve in conjunction with aspirin 75 mg daily. Additional medications included atorvastatin for cardiovascular risk reduction, metoprolol for rate control and cardioprotection, and amlodipine for blood pressure optimization. Comprehensive counseling was provided regarding warfarin-related dietary restrictions (particularly vitamin K-containing foods), potential drug interactions, the importance of INR monitoring, and lifestyle modifications necessary for safe long-term anticoagulation management.

At the six-week follow-up, the patient remained completely asymptomatic with resolution of palpitations and chest vibrations. He had resumed light physical activity with plans for gradual escalation under cardiology supervision. INR levels were therapeutic and stable, and the patient demonstrated excellent understanding and compliance with anticoagulation protocols. He expressed satisfaction with the surgical outcome and adapted well to the mechanical valve, including the audible clicking sound associated with valve function.

## Discussion

BAV predisposes young adults to early and accelerated valvular dysfunction, with severe AS and AR frequently requiring surgical intervention before the fourth decade of life [1,11]. The management of young patients with BAV presents unique challenges that extend beyond the immediate perioperative period, as the choice of surgical approach significantly impacts long-term outcomes, quality of life, exercise capacity, and the potential need for future reinterventions [2,12].

Surgical options and decision-making process

The Ross procedure has traditionally been considered the gold standard for young patients with BAV requiring aortic valve replacement. This technique involves replacing the diseased aortic valve with the patient's own pulmonary valve (autograft), followed by replacement of the pulmonary valve with a homograft [13]. The theoretical advantages of the Ross procedure include excellent hemodynamic performance, potential for growth in pediatric patients, absence of anticoagulation requirements, and superior long-term durability compared to bioprosthetic valves [3,14].

However, several studies have reported varying outcomes with the Ross procedure, particularly regarding long-term autograft durability. El-Hamamsy et al. demonstrated excellent 20-year outcomes in a randomized controlled trial, with 95% freedom from autograft valve-related mortality and 85% freedom from autograft reintervention [4]. Conversely, other series have reported higher rates of autograft dysfunction, particularly in patients with BAV and those with aortic regurgitation as the primary pathology [15,16].

The technical success of the Ross procedure is highly dependent on appropriate patient selection and anatomical considerations. Significant annulus mismatch, as encountered in our patient (aortic annulus 30 mm vs. pulmonary annulus 24 mm), represents a relative contraindication due to increased risk of autograft dysfunction and technical failure [4,17].

Significant annular size mismatch between the aortic and pulmonary roots is independently associated with increased risk of long-term autograft failure, as the undersized pulmonary autograft exposed to systemic pressures undergoes progressive dilatation leading to aortic regurgitation and potential reoperation [18]. The size discrepancy would necessitate either autograft manipulation or implantation of an oversized valve, both of which compromise long-term durability.

Mechanical valve replacement in young patients

Mechanical valve replacement represents a well-established and durable option for young patients with valvular heart disease. Traditional mechanical valves, including the St. Jude Medical (St. Jude Medical, Inc., Saint Paul, Minnesota, United States) and Medtronic-Hall (Medtronic plc, Minneapolis, Minnesota, United States) valves, have demonstrated excellent long-term durability with actuarial patient survival rates of approximately 41-58% at 15 years and 17-28% at 20-25 years, with these survival rates being primarily determined by patient-related factors rather than valve failure [19]. However, the requirement for lifelong anticoagulation with vitamin K antagonists has been a significant limitation, particularly in young, active patients.

The On-X mechanical valve, utilized in this case, represents a significant advancement in mechanical valve technology. The valve features a unique design with pyrolytic carbon leaflets and a titanium housing, engineered to optimize hemodynamics and reduce thrombogenicity. Several clinical studies have demonstrated the superior performance characteristics of the On-X valve compared to earlier-generation mechanical valves [20].

The PROACT (Prospective Randomized On-X Anticoagulation Clinical Trial) study, a landmark FDA-approved randomized controlled trial, demonstrated that patients with On-X aortic valves who had elevated risk factors for thromboembolism could be safely managed with reduced anticoagulation intensity (INR 1.5-2.0) when combined with aspirin 81 mg daily [5]. This reduced anticoagulation requirement represents a significant advantage for young, active patients, potentially reducing bleeding complications while maintaining effective stroke prevention.

Long-term outcomes data for the On-X valve have been encouraging [21]. Additionally, the valve demonstrated excellent hemodynamic performance with low transvalvular gradients and minimal patient-prosthesis mismatch, factors that are particularly important for maintaining exercise capacity in young, athletic patients.

Comparison with bioprosthetic valves

Bioprosthetic valves were considered in this case but deemed less suitable due to their limited durability in young patients. Contemporary tissue valves, including bovine pericardial and porcine valves, typically demonstrate structural valve deterioration within 10-15 years when implanted in patients under 40 years of age [22,23]. The accelerated degeneration in younger patients is attributed to higher cardiac output, increased physical activity, and more active calcium metabolism.

Recent studies have shown some improvement in bioprosthetic valve durability with newer generation valves. However, even with improved durability, the likelihood of requiring valve reintervention during a young patient's lifetime remains high, making mechanical valves more appropriate for this population [24].

The concept of valve-in-valve transcatheter aortic valve replacement (TAVR) has emerged as a potential solution for failed bioprosthetic valves, potentially making tissue valves more attractive for younger patients. However, long-term data on valve-in-valve TAVR outcomes remain limited, and concerns regarding higher gradients and reduced effective orifice area persist.

Anticoagulation considerations

The requirement for lifelong anticoagulation represents the primary disadvantage of mechanical valve replacement, particularly in young, active patients. Traditional anticoagulation targets (INR 2.5-3.5 for aortic mechanical valves) carry significant bleeding risks, with annual rates of major bleeding ranging from 1% to 3% per year. These risks are particularly concerning in athletic individuals who participate in contact sports or activities with inherent trauma risk.

The reduced anticoagulation intensity achievable with the On-X valve (INR 1.5-2.0) potentially reduces bleeding complications while maintaining adequate thromboembolic protection. However, this approach requires careful patient selection and excludes patients with atrial fibrillation, previous thromboembolism, or hypercoagulable states. Long-term monitoring and patient education remain essential components of successful anticoagulation management.

Direct oral anticoagulants (DOACs) have revolutionized anticoagulation therapy in many clinical scenarios but remain contraindicated in patients with mechanical heart valves. The RE-ALIGN (Randomized Evaluation of Long-Term Anticoagulation Therapy) trial, which evaluated dabigatran in patients with mechanical valves, was terminated early due to increased rates of thromboembolism and bleeding complications compared to warfarin [25].

Activity restrictions and quality of life

Young patients with mechanical valves face important decisions regarding physical activity and lifestyle modifications. Current guidelines recommend avoiding contact sports and activities with a high risk of trauma due to bleeding concerns. However, many patients successfully maintain active lifestyles with appropriate precautions and regular monitoring.

Studies evaluating quality of life outcomes in young patients with mechanical valves have generally shown favorable results. Non-elderly adult patients (ages 18-60) after aortic valve replacement reported good perceived quality of life, with physical health scores comparable to or slightly below the general population, but interestingly better mental health scores [26].

Case-specific considerations

This case illustrates several important aspects of managing young patients with BAV. The patient's medical background facilitated understanding of the treatment options and compliance with anticoagulation protocols, potentially mitigating some concerns associated with warfarin therapy. His asymptomatic presentation despite severe AS highlights the insidious nature of BAV-related complications and emphasizes the importance of early detection through careful clinical examination.

The anatomical constraints that precluded the Ross procedure underscore the importance of comprehensive preoperative evaluation and individualized treatment planning. The excellent short-term outcomes achieved with the On-X valve, including low transvalvular gradients and preserved ventricular function, support its use in young patients with BAV.

Long-term follow-up will be essential to monitor for potential complications, including thromboembolism, bleeding, structural valve deterioration, and endocarditis. While routine annual echocardiography is not typically required for normally functioning mechanical valves in asymptomatic patients with no coexistent pathology, lifelong cardiology follow-up is essential in a specialist multidisciplinary valve clinic to monitor for clinical changes, assess anticoagulation management, and perform echocardiography when clinically indicated. Exercise testing may be beneficial to optimize activity recommendations and ensure an appropriate cardiovascular response to exercise.

## Conclusions

This case demonstrates the successful management of severe aortic stenosis and moderate regurgitation in a 28-year-old athletic physician with a bicuspid aortic valve using On-X mechanical valve replacement. The significant annulus mismatch (aortic annulus 30 mm versus pulmonary annulus 24 mm) that precluded the Ross procedure underscores the critical importance of comprehensive anatomical assessment in surgical planning. Despite the patient's initial preference for avoiding lifelong anticoagulation, the anatomical constraints necessitated a mechanical valve approach, which proved highly successful with excellent hemodynamic outcomes, including a postoperative peak gradient of only 11 mmHg and preserved ejection fraction of 55%.

The On-X mechanical valve demonstrated several advantages in this young, active patient, including superior hemodynamic performance and the potential for reduced anticoagulation intensity (INR 1.5-2.0 with aspirin). However, this case highlights that successful outcomes in young BAV patients require individualized, multidisciplinary treatment planning that accounts for anatomical constraints, lifestyle considerations, and long-term durability needs. The patient's medical background facilitated understanding of anticoagulation protocols, but all young patients receiving mechanical valves require comprehensive counseling regarding lifelong monitoring, lifestyle modifications, and activity restrictions. Long-term follow-up with annual echocardiography and regular INR monitoring remains essential to evaluate valve function and identify potential complications.
